# Plant Volatiles of Lettuce and Chicory Cultivated in Aquaponics Are Associated to Their Microbial Community

**DOI:** 10.3390/microorganisms9030580

**Published:** 2021-03-12

**Authors:** Lorenzo Nissen, Flavia Casciano, Andrea Gianotti

**Affiliations:** 1 CIRI—Interdepartmental Centre of Agri-Food Industrial Research, *Alma Mater Studiorum*—University of Bologna, P.za G. Goidanich, 60, 47521 Cesena, Italy; lorenzo.nissen@unibo.it; 2 DiSTAL—Department of Agricultural and Food Sciences, *Alma Mater Studiorum*—University of Bologna, P.za G. Goidanich, 60, 47521 Cesena, Italy; flavia.casciano2@unibo.it

**Keywords:** *Lactuca sativa*, *Chicorium intybus*, VOCs, One Health, Nile tilapia, holobiome, food safety

## Abstract

In this work, an aquaponic cultivation system for *Lactuca sativa* (L.) and *Chicorium intybus* (L.) was compared to a hydroponic one, focusing on the main microbial populations related to food safety and their volatile compounds (VOCs), concluding with Spearman correlations among the microbes and VOCs. Different sections of both systems were sampled at the end of the commercial development of the plants. Plants cultivated in aquaponics were in general more contaminated than those from hydroponics, while for the cultivation waters a higher contamination of the hydroponics than aquaponics system was unexpectedly observed. Furthermore, the chicory exhibited higher levels of all microbial groups compared to lettuce grown under the same cultivation system. The results obtained also showed correlations between the distribution of some VOCs and microbial groups in the phyllosphere, while some examples of positive correlations between 2-nonanone (a positive phytostimulant compound) and anaerobic bacilli of the rhizosphere in lettuce were reported. So far, multivariate analysis of VOCs was able to discriminate on the basis of varieties but not on the cultivation systems. In conclusion, the microbial characteristics of the two ecosystems depended both on plant variety and cultivation method but further studies will need to deeply investigate the variables influencing the microbial quality of vegetable foods obtained by aquaponics. On the other hand, the analysis of the VOCs was more related to the microbial community of each plant variety considered, whatever the cultivation system. In precision agriculture, metabolomics may represent an opportunity to study the holobiome and through it the interactions between plants and their microbial populations, to possibly provide for a tool to assess the microbiological quality of vegetable foods obtained by aquaponic systems.

## 1. Introduction

On a global level, society is faced with a huge challenge—being able to provide food to an ever-growing population and conserve the environment and natural resources. Rising food demand has already put a strain on natural resources, resulting in soil erosion, biodiversity loss and pollution around the world, presenting new challenges in food security and sustainable food production [1]. To address these challenges, precision agriculture, sustainable food production and environmental protection are fundamental and will require a One Health approach, according to which human, animal and environmental health are inextricably linked. This approach can be applied to increase sustainable agricultural practices and improve the health and general well-being of humans, animals and the environment. One form of sustainable agricultural practice is aquaponics. Aquaponics is a system that combines hydroponic cultivation with aquaculture and allows the recycling of water from the fish farm, particularly rich in nutrients, which is used as an organic fertilizer for plants grown in the hydroponic system [2]. The use of this system involves a lower environmental impact thanks to the absence of mineral fertilizers and the discharge into the environment of aquaculture waste [3]. Aquaponics is creating more and more space for commercial and scientific applications, and although there are studies that described the microbiota related to the efficiency of the system [3,4,5,6,7], knowledge of the microbial ecology related to the food quality of aquaponic plants, and of the system, is scarce. An important component of an aquaponic system is the microbial ecology, responsible in particular for the nitrification process. In fact, fish produce nitrogen in the form of ammonia, which must be converted from nitrite to nitrate, which can be used by plants [8]. The microbial component, however, plays an important role in the balance of the aquaponic system, which goes far beyond the nitrification process alone. It has been found, for example, that plant growth-promoting microbes (PGPM) also play a role in nutrient absorption [9] and could thus be responsible for the high yields of plants grown in aquaponics, comparable to those obtained with hydroponic cultivation although the nutrient level is significantly lower [10]. Furthermore, the microbial population of this cultivation system is of paramount importance for a safety assessment of the aquaponics-derived food, which still represent a concern for food safety policy makers. Considering this aspect is of fundamental importance to study the volatilome (the composition of volatile organic compounds VOCs) of the cultivation system as well as that of the plants, and by highlighting the possible correlations among the microbes and the VOCs, one can assess a particular microbial group regarding its production of detrimental (toxic) or beneficial (phytostimulant) compounds. Several studies have used metabolomics to explore the impact of different cultivation techniques on plant products and to evaluate the quality of the latter [11,12,13,14,15]. Therefore, the study of the metabolome of plants grown in aquaponics and hydroponics is interesting to identify any molecules characterizing one or the other cultivation method and to indirectly characterize the microflora. In this work, we characterized the microbial ecology of an aquaponic system and a hydroponic system by comparing two varieties of leafy vegetables grown in both systems. The aim of this study was to explore the microbial communities of plants grown in aquaponics and to evaluate their microbiological safety and flavor profile by comparing the plants varieties grown in both hydroponics and aquaponics. Besides, Spearman correlations between the microbes and VOCs allowed us to address the specific contribution of the microbes to VOC production.

## 2. Materials and Methods

### 2.1. Sample Collection and Preparation

For this study, the “MAEVA” aquaponic system from the company IRCIPONIC (IRCI S.p.a., Pietracuta, Rimini, Italy) was used. The system consisted of an animal breeding tank and a plant growth bed. The breeding tank, which was a recirculating aquaculture system, was equipped with an automatic mechanical filtering system and an oxygenation system for the water of the tank in which the fish swam. The plant growth bed consisted of “floating beds” in which the vegetable species to be grown were planted. The sampling was carried out in the company, collecting a duplicate of samples for each ecological niche to be analyzed. The samples were taken after 30 days of plant development. In particular, the following samples were collected: (I) 50 mL of water from the biofilter; (II) 50 mL of fish tank water; (III) 50 mL of cultivation water from the aquaponic system; (IV) 50 mL of cultivation water from the hydroponic system; (V) a specimen of tilapia of about 4 months of development; (VI) *Lactuca sativa* var. Salanova with roots grown in aquaponic system; (VII) *Lactuca sativa* var. Salanova with roots grown in hydroponic system; (VIII) *Cichorium intybus* with roots grown in aquaponic system; and (IX) *Cichorium intybus* with roots grown in a hydroponic system. The sample codes’ descriptions can be found in Table S1.

### 2.2. Plants Cultivation

Lettuce (*Lactuca sativa*) var. Salanova (Ortomio, Forlì, Italy) and *Cichorium intybus* (Ortomio, Italy) were sown in non-woven fabric (TNT) cubes and placed in a phytotron with a day/night photoperiod of 18 h/6 h, a temperature of 15 °C and a relative humidity of 70%. The lighting system consisted of two LED panels. Plugs were transplanted into hydroponic boxes that mimic the floating bed system and the aquaponic growing tank was connected to the tank with tilapia fish. The aquaponic cultivation tank and the fish tank had measures of 1.23 × 1.23 × 0.20 m and were filled with 1 m^2^ of water, while the hydroponic tank had measures equal to 1.23 × 1.23 × 0.20 m and was filled with 0.4 m^2^ of water. Plants were grown for 30 days.

### 2.3. Microbial Quantification by Culture-Dependent Method

Microbial counts were made at the end of the plants’ cultivation cycle (30 days), soon after the samples were collected at the cultivation greenhouse and transported at 4 °C to the laboratory, adapting the ISO methodologies (Table S2). Briefly, liquid samples were directly diluted in pre-sterilized physiological solution (NaCl 0.9 % *w/v*) tubes to obtain a series of decimal dilutions, while the solid samples were homogenized in a 1:10 (*w/v*) dilution for 2 min in a Stomacher 3500 paddle blender (Seward Ltd., Worthing, UK), and from that on a series of decimal dilution tubes was prepared. Solid samples were homogeneously selected from different parts of the specimen. To enumerate mesophilic aerobic bacteria, mesophilic anaerobic bacteria, psychrotrophic bacteria, enterococci, molds and yeasts, and *Pseudomonas* spp., 0.1 mL aliquots of the dilution tubes were spread by spatulation on the respective selective agar media. To enumerate the *Enterobacteriaceae*, coliforms and lactic acid bacteria (LAB), 1 mL aliquots were transferred into empty petri capsules and subsequently a double layer of agar was poured over. The selective media and incubation conditions for each microbial target are described in Table S2.

### 2.4. Solid-Phase Microextraction–Gas Chromatography–Mass Spectrometry (SPME-GC-MS)

Evaluation of the volatile organic compounds (VOCs) was carried out on an Agilent 7890A Gas Chromatograph (Agilent Technologies, Santa Clara, CA, USA) coupled to an Agilent Technologies 5975 mass spectrometer operating in the electron impact mode (ionization voltage of 70 eV), equipped with a Chrompack CP-Wax 52 CB capillary column (50 m length, 0.32 mm ID) (Chrompack, Middelburg, Nederlands). The SPME-GC-MS (solid phase micro-extraction–gas chromatography–mass spectrometry) protocol and the identification of volatile compounds were done according to previous reports, with minor modifications [16,17]. Briefly, once samples were received, 3 g or mL of solid or liquid samples were aseptically transferred onto a 10 mL borosilicate glass GC/Headspace vial (LLG GmbH, Meckenheim, Germany), the cap was sealed with a ferrule and the vials eventually stored at −80 °C. The solid samples were homogeneously selected from different parts of the specimen. Prior analyses 6 μL of 10,000 ppm of 2-pentanol, 4-methyl (Sigma-Aldrich, St. Louis, MO, USA) were injected into the vial and let to equilibrate for 10 min at 40 °C in a water bath. The SPME fiber was then exposed to each sample for 40 min at 40 °C, and finally the fiber was inserted into the injection port of the GC for a 10 min sample desorption. The temperature program was 50 °C for 0 min, then programmed at 1.5 °C/min to 65 °C and finally at 3.5 °C/min to 220 °C, which was maintained for 20 min. The injector, interface and ion source temperatures were 250, 250 and 230 °C, respectively. Injections were carried out in splitless mode, and helium (3 mL/min) was used as carrier gas. Before each head space sampling, the fiber was exposed to the GC inlet for 10 min for thermal desorption at 250 °C in a blank sample. Identification of molecules was carried out by comparing their retention times with those of pure compounds (Sigma-Aldrich, USA) and confirmed by searching mass spectra in the available databases, namely, NIST 11 MSMS library (NIST, Gaithersburg, MD, USA) and Wiley Registry 8th Edition (John Wiley & Sons, Inc, Hoboken, NJ, USA). 

### 2.5. Statistical Analyses

All statistical analyses were performed using TIBCO Statistica 8.0 (Tibco Inc., Palo Alto, CA, USA). Normality was checked with the Shapiro–Wilk test, while homoscedasticity was evaluated with Levene’s test [18]. Differences between all samples were evaluated with untargeted Analysis of Variance (ANOVA), set at *p* < 0.05, and a quantification heatmap on the relative abundances of the VOCs was generated for a broad description of the VOC profiles of the sampling sites at the end of commercial development of the plants (30 days). Untargeted multivariate analysis employing Principal Component Analysis (PCA) and K-mean clustering were used to discriminate samples by descriptors. Multivariate ANOVA (MANOVA), set at *p* < 0.01, was used to weigh and address the contributions of the descriptors. Spearman rank correlations, Pearson cluster analysis and a two-way joining heatmap were used to study the relationship between the variables. For the post-hoc testing, an HSD Tukey’s test was employed (*p* < 0.05). An independently normalized dataset was proposed for each ecosystem set of molecules. The data were normalized using the mean centering method.

## 3. Results and Discussion

### 3.1. General Aspects of the Volatilome Analysis through SPME-GC-MS

**Figure 1:**
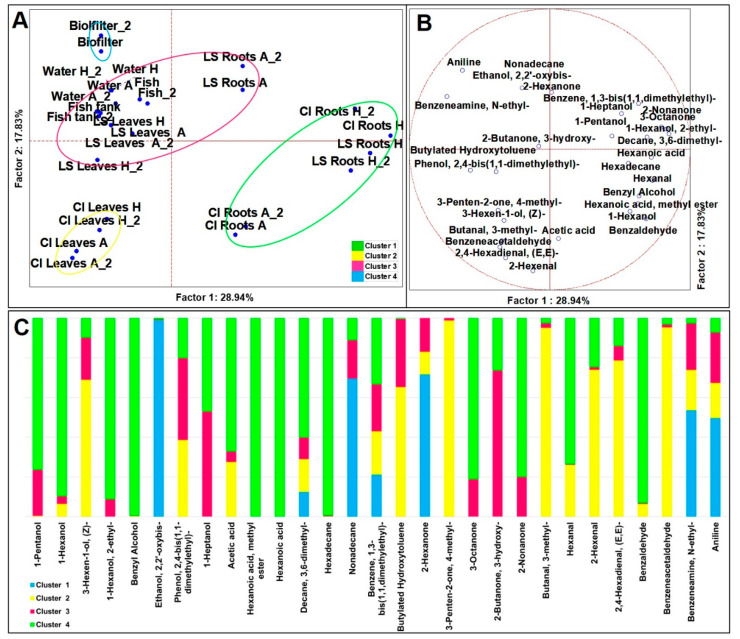
Principal Component Analysis (PCA) of cases (**A**) and variables (**B**) on VOCs; (**C**) K-means cluster analysis (at least *p* < 0.05).

Through SPME-GC-MS, among the 13 duplicated cases (*n* = 26), 45 molecules were identified with a more than 80% of similarity with the NIST 11 MSMS library and the NIST MS Search program 2.0 (NIST, USA). On average, 24 were relatively quantified in the system, 32 in the leaves and 37 in the roots (Figure S1). For a landscape description of the volatilome, a dataset of 29 molecules was generated, and to find evidence that those compounds able to discriminate among the ecological niches, we chose to super-normalize the VOC datasets on the basis of the sampling sites (*n* = 26) (Table S1) in these three ecosystems: (i) *Lactuca sativa* (LS) (*n* = 8); (ii) *Cichorium intybus* (CI) (*n* = 8); and (iii) the cultivation systems (*n* = 10). So far, to each dataset we applied multivariate analyses, as untargeted PCA (Principal Component Analysis) (*p* < 0.05), to weight the descriptors, and targeted MANOVA (*p* < 0.01) (Figure S2) to address the specific contributions to VOC production [19,20,21]. Twenty-nine significant VOCs over 13 duplicate cases (ANOVA, *p* < 0.05) were processed by the PCA, which grouped the samples in different directions on the plane where K-means analysis identified four clusters (Figure 1A–C). Two clusters were made only by roots and leaves, one by both leaves and roots and the system components, and one by the biofilter. Cluster 1 was positioned on Quadrant I and included only the biofilter. It was described by lower speciation made by just 7 compounds, including nonadecane and 2-hexanone, as more abundant than in the other clusters, and ethanol, 2,2′-oxybis as an exclusive signature. Cluster 2 contained only CI leaves, from both the aquaponic and hydroponic system. It was described by 17 variables and it was addressed by higher concentrations of 3-penten-2-one, 4-methyl-, butanal, 3-methyl-, and benzeneacetaldehyde. In fact, from the MANOVA (Figure S2), the first compound accounted for 93.1% of total cases to CI leaves, the second accounted for 86.9%, while the third to 89.7%. In the literature, it is known that butanal, 3-methyl is produced both from the plant tissue and microorganisms and is responsible for off-odors with a fermentative/acidic character [22]. The presence of this molecule in CI leaves could be due to the shear-induced stress. Cluster 3 included components of the system and leaves, and roots of LS. This cluster was described by 22 variables, and amid 2-butanone, 3-hydroxy- (also known as acetoin) was the most abundant among every cluster. Some microorganisms, such as LAB, have the ability to synthesize acetoin using different enzymes and pathways [23]. Natural acetoin also has been detected in fruits, vegetables and flours, contributing to their distinct natural flavors [23], and has shown to be an antimicrobial targeting foodborne pathogens and spoilage microorganisms [24]. Cluster 3 was described also by 1-heptanol. In fact, from the MANOVA (Figure S2), this compound accounted for 81.7% of the total cases to LS roots. Lastly, Cluster 4 was positioned oppositely to Clusters 1 and 2, and it was formed by all the roots, except LS from aquaponics. It was described by a higher speciation, made by 27 molecules out of 29 and by a typical signature made by benzyl alcohol, hexanoic acid, methyl ester-, hexanoic acid and hexadecane.

### 3.2. Volatilome Analysis of Lactuca Sativa

**Figure 2:**
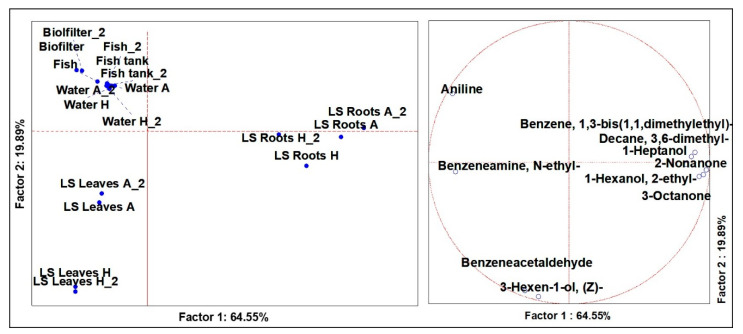
Principal component analysis (PCA) of the cases (**left**) and variables (**right**) of the significant (ANOVA *p* < 0.05) VOCs of *Lactuca sativa*.

To better observe the differences in the two varieties, normalization of the dataset and statistical analysis were performed for LS, CI and for the system. From the analysis of variance, including the LS samples (*n* = 18), significant differences (*p* < 0.01) were defined for 10 different molecules. From the PCAs (Figure 2), a robust plane was evidenced, based on two factors defining 19.89% and 64.55% of the total representation. Results from the PCA allowed to discriminate the molecules that characterized the leaves, the roots and the system, while no differences were found between the plants grown in aquaponics rather than hydroponics. In particular, aniline was the molecule that described the system, while benzeneamine, N-ethyl-, benzeneacetaldehyde and 3-hexen-1-ol, (Z)- were descriptors of LS leaves, either from aquaponic or hydroponic systems. Benzeneacetaldehyde is a secondary VOCs considered to be the product of lipid oxidation, which results in off-odors [25]. This molecule derives from acetaldehyde as a result of anaerobic metabolism, which increases under stress conditions. Lastly, the roots were described by 6 VOCs among which one, namely 2-nonanone, is particularly interesting. Actually, 2-nonanone is reported to be a phytostimulant effective for root elongation and differentiation, and is produced by anaerobic bacteria of the rhizosphere, e.g., *Bacillus* spp. [26].

### 3.3. Volatilome Analysis of Cichorium Intybus

**Figure 3:**
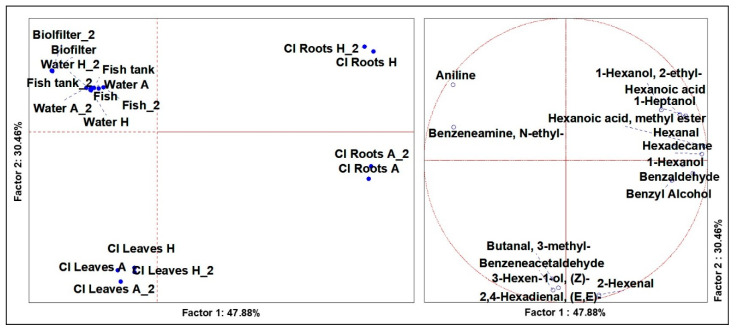
Principal Component Analysis (PCA) of the cases (**left**) and variables (**right**) of the significant (ANOVA *p* < 0.05) VOCs of *Chicorium intybus*.

From the analysis of variance, including the CI samples (*n* = 18), significant differences (*p* < 0.01) were defined for 16 VOCs. From the PCAs (Figure 3), a robust plane was evidenced, based on two factors defining 30.46% and 47.88% of the total representations. Similar to the results obtained for the LS samples, PCA discriminated the molecules that characterized the three different ecosystems, which were leaves, roots and system components. However, the cases related to roots from aquaponics and hydroponics cultivations were set distant to each other in different diagram quadrants. In the first case, in fact, the roots were described by 1-hexanol, benzaldehyde and benzyl alcohol, while in the second case roots were described by 1-hexanol, 2-ethyl-, hexanoic acid and 1-heptanol. In the literature, it is known that benzaldehyde, commonly detected in plant VOC profiling, plays a key role in plant fitness. In plants, benzaldehyde biosynthesis is a product from trans-cinnamic acid, which is produced by the breakdown of phenylalanine [27]. Benzaldehyde and benzyl alcohol (probably derived from benalzaldehyde by an oxidoreductase) are found to be accumulated in cucumber roots and flowers [27]. The VOCs describing CI leaves were in common with LS leaves, notably benzeneacetaldehyde and 3-hexen-1-ol, (Z)-, but the CI leaves were characterized by a higher number of VOCs, including also butanal, 3-methyl-, 2-hexenal and 2,4-hexadienal, (E, E)-. Green aldehydes are the compounds responsible for the green aromatic note. They are produced by linoleic acid in injured plant tissues when the cell membrane is damaged, and many enzymes are thus released [28]. Following the damage to the cell membrane, free linoleic acid and linoleic hydroperoxides are released. The action of lyase on hydroperoxides lead to the formation of 2-hexenal, a compound responsible for the natural green aroma and with a fungistatic action in the gaseous phase [28]. 

### 3.4. Volatilome Analysis of the System

**Figure 4:**
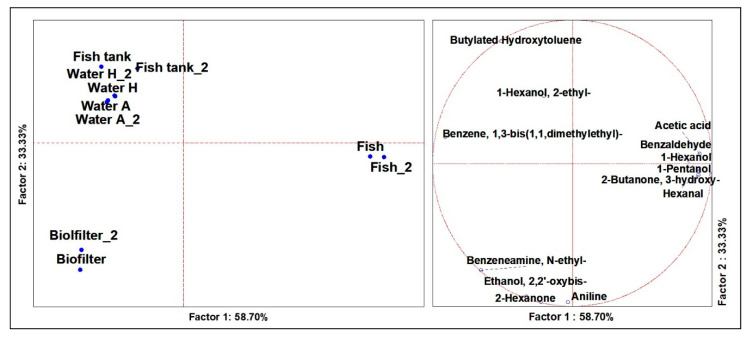
Principal Component Analysis (PCA) of the cases (**left**) and variables (**right**) of the significant (ANOVA *p* < 0.05) VOCs of the cultivation system.

From the ANOVA, including samples obtained from different sensible sites of the cultivation systems (*n* = 13), significant differences (*p* < 0.01) were defined for the 16 different VOCs. From the PCAs (Figure 4), a robust plane was evidenced, based on two factors defining 33.33% and 58.70% of the total representations. Results from the PCA showed a clear distinction amid biofilters, fishes, and waters. In particular, the waters were better described by butylated hydroxytoluene, 1-hexanol, 2-ethyl- and benzene, 1,3-bis (1,1-dimethylethyl)-, while the biofilter was characterized by benzeneamine, N-ethyl-, ethanol, 2,2′-oxybis-, 2-hexanone and aniline. The attribution of aniline to the biofilter is desirable, ensuring that this molecule did not flow into the cultivation waters, because it has a strong toxicity and would inhibit the growth of aquatic plants and animals [29]. Finally, fishes were described by 2-butanone, 3-hydroxy-, hexanal and 1-pentanol.

### 3.5. Microbial Quantification

Thirteen different ecological niches were sampled (Table S1) to target nine different microbial groups (Table S2), in order to define the core microbiota inhabiting or contaminating the plants and cultivation environment after 30 days of development. The microbial groups sought were coliforms, *Enterobacteriaceae*, enterococci, LAB, *Pseudomonas* spp., aerobic mesophilic bacteria, anaerobic mesophilic bacteria, psychrotrophic bacteria and yeasts and molds. Again, the results will be presented splitting the outputs based on ecosystems, namely, the leaves, roots and the cultivation apparatus. For this latter, a dataset of the baseline quantification values (before to put to abode commercial young seedlings) is supplied in the Supplementary Materials (Table S3). Generally, mean loads of 3.91, 5.37 and 3.74 Log_10_ CFU/mL were quantified in the different cases related to leaves, roots and the cultivation system, respectively.

#### 3.5.1. Microbial Quantification of the Phyllospheres

**Figure 5:**
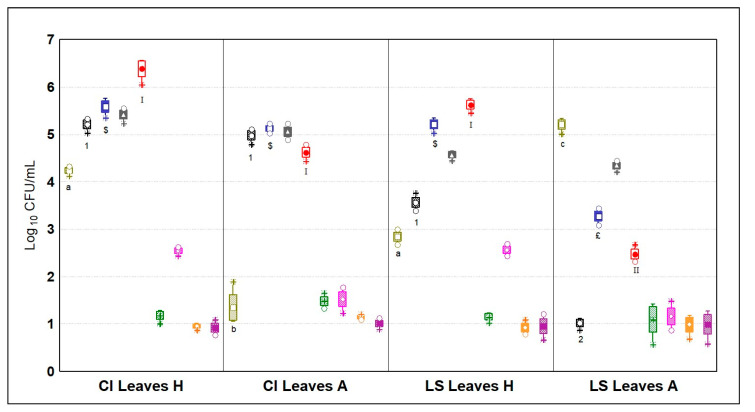
Microbial quantities in Log_10_ CFU/mL (*n* = 4) of the nine different microbial targets; olive green = coliforms; black stripes = *Enterobacteriaceae*; blue = aerobic mesophilic bacteria; gray = anaerobic mesophilic bacteria; red = psychrotrophic bacteria; green = lactic acid bacteria; fuchsia = yeasts and molds; orange = *Pseudomonas* spp.; violet = enterococci; CI = *Chicorium intybus*; H = hydroponic cultivation; A = aquaponic cultivation; LS = *Lactuca sativa*. Different letters, numbers, Latin numbers, or symbols indicate statistical significance among a microbial target by Tukey’s HSD test (*p* < 0.05). Samples were analyzed in duplicate from two independent experiments (*n* = 4). Box = mean; rectangles = mean*S.D.; whiskers = min and max values; asterisks = extremes; dots = outliers.

Quantification performed on leaves at the end of the cultivation period of the plants (Figure 5) had the lowest loads with respect to the three ecosystems (i.e., leaves, roots and cultivation system). Leaves had high concentrations of mesophilic and psychrotrophic bacteria, accounting for a mean value of 4.85 ± 0.14 Log_10_ CFU/mL. In particular, the most abundant bacterial group that was found in the leaves samples (*n* = 8) was that of psychrotrophic bacteria in CI leaves in the aquaponic system, which was 1.90 Log_10_ CFU/mL higher than that in the hydroponic system (*p* < 0.05). Lower values, but with a similar trend, were recorded on LS leaves; otherwise, here the difference between the two cultivation systems was larger (3.24 Log_10_ CFU/mL) (*p* < 0.05). Other authors have reported a mean abundance around 4.81 Log_10_ CFU/mL of psychrotrophic bacteria on iceberg lettuce leaves traditionally cultivated after 1 day of storage, surging up to 8.26 Log_10_ CFU/mL after 10 days of storage in a protective atmosphere [22]. Since these loads are causative agents of spoilage on vegetables [30], a lower contamination of this microbial group prior to storage, as we reported for LS growing in an aquaponic system, is important to maintain a proper shelf life. The second most abundant bacterial group was that of the *Enterobacteriaceae*, accounting on average for 3.46 Log_10_ CFU/mL, reaching the top in CI leaves (5.14 ± 0.16 Log_10_ CFU/mL) with no difference between the two systems (*p* > 0.05), but 2.27-fold higher than in LS leaves (*p* < 0.05). In particular, less than 1 Log_10_ CFU/mL was quantified in LS leaves of plants grown hydroponically. Interestingly, coliforms quantified in leaves had the opposite distribution; in fact, they were on average 1.93 Log_10_ CFU/mL more abundant on LS than in CI leaves, but the former had higher loads in the aquaponic system while the latter in the hydroponic system. On iceberg lettuce cultivated in soil, *Enterobacteriaceae* quantification had a mean value of 3.54 Log_10_ CFU/mL but reached up to 6.71 Log_10_ CFU/mL after ten days of protective storage, becoming risky for consumption [22]. Again, the outputs that we obtained indicate that aquaponic cultivation of LS is safer than in soil cultivation.

#### 3.5.2. Microbial Quantification of the Rhizospheres

**Figure 6:**
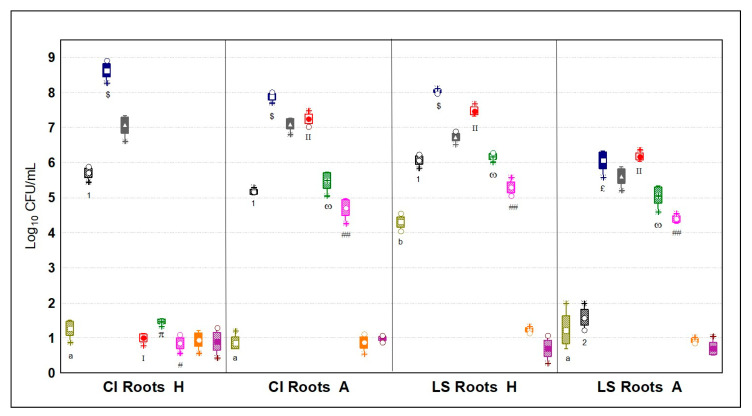
Microbial quantities in Log_10_ CFU/mL (*n* = 4) of nine different microbial targets; olive green = coliforms; black stripes = *Enterobacteriaceae*; blue = aerobic mesophilic bacteria; gray = anaerobic mesophilic bacteria; red = psychrotrophic bacteria; green = lactic acid bacteria; fuchsia = yeasts and molds; orange = *Pseudomonas* spp.; violet = enterococci; CI = *Chicorium intybus*; H = hydroponic cultivation; A = aquaponic cultivation; LS = *Lactuca sativa*. Different letters, numbers, Greek letters, Latin numbers or symbols indicate statistical significance among a microbial target by Tukey’s HSD test (*p* < 0.05). Samples were analyzed in duplicate from two independent experiments (*n* = 4). Box = mean; rectangles = mean*S.D.; whiskers = min and max values; asterisks = extremes; dots = outliers.

The rhizosphere of the plants was the ecosystem with the highest mean load of microorganisms. Top concentrations were scored for both aerobic and anaerobic mesophilic bacteria (7.35 ± 0.79 Log_10_ CFU/mL) followed by psychrotrophic bacteria, which were 2.51 and 2.23 Log_10_ CFU/mL more than in leaves. Interestingly, this ecosystem was the richest in yeast and mold abundances, hitting a mean value of 5.04 ± 0.06 Log_10_ CFU/mL, or rather, 2.39-fold more than the runner up, i.e., leaves (Figure 6). Significant differences among the roots samples from the cultivation systems were found only for LS, where the aquaponic overwhelmed with 1.33 ± 0.15 Log_10_ CFU/mL (*p* < 0.05). In particular, LS roots had top values in coliforms and *Enterobacteriaceae*; those in the aquaponic system were 2.22- and 3.02-fold more abundant. Literature reports on the microbial characterization of lettuce roots in soilless systems are few and any comparisons appear slippery; nevertheless, a study observed that almost 60% of the microbiota of roots of lettuce cultivated in an aquaponic system was composed of Proteobacteria [31], to which *Enterobacteriaceae* belong. Roots were characterized by a larger microbial diversity; in fact, besides the other microbial groups, yeasts and molds were abundantly quantified just in this ecosystem, with a mean value of 4.03 ± 2.06 Log_10_ CFU/mL, as well as lactic acid bacteria 4.67 ± 2.13 Log_10_ CFU/mL, with the exception of fish fillets. These two microbial groups had either top levels in LS roots from aquaponic plants and bottom levels in CI roots from the same cultivation method. These interactions are renown to happen in soil environments, but limited are the studies on aquaponics [32]. Along with many bacilli, different species from the yeast *Streptomyces* spp., likewise different species of *Enterobacter* spp., are considered PGPM, which establish a symbiosis within the rhizosphere and improve the nutrient uptake of plants, as well as serving as a natural defense producing antimicrobial compounds against root phytopathogens [31,33].

#### 3.5.3. Microbial Quantification of the Cultivation Systems

**Figure 7:**
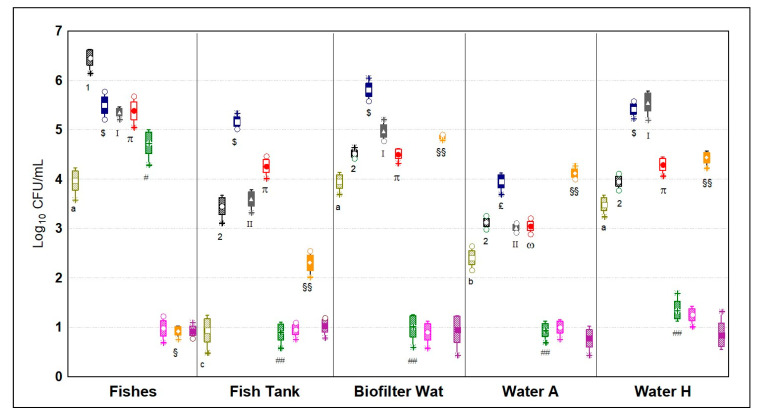
Microbial quantifications in Log_10_ CFU/mL (*n* = 4) of the nine different microbial targets; olive green = coliforms; black stripes = *Enterobacteriaceae*; blue = aerobic mesophilic bacteria; gray = anaerobic mesophilic bacteria; red = psychrotrophic bacteria; green = lactic acid bacteria; fuchsia = yeasts and molds; orange = *Pseudomonas* spp.; violet = enterococci; Fishes = Fillets of Nile tilapia; Fish tank = water samples from tank for fishes; Biofilter Wat = water samples from the biofilter; Water A = water samples from tank of aquaponic cultivation; Water H = water samples from tank of hydroponic cultivation. Different letters, numbers, Latin numbers, Greek letters, or symbols indicate statistical significance among a microbial target by Tukey’s HSD test (*p* < 0.05). Samples were analyzed in duplicate from two independent experiments (*n* = 4). Box = mean; rectangles = mean*S.D.; whiskers = min and max values; asterisks = extremes; dots = outliers.

The ecological niches related to the cultivation ecosystem (Figure 7) recorded the highest mean concentration of microorganisms, which had a mean value of 3.93 ± 1.78 Log_10_ CFU/mL among the nine targets assayed. In detail, this site had top abundances of *Enterobacteriaceae*, scoring in the fish fillets the highest value among every case tested (6.59 ± 0.22 Log_10_ CFU/mL). Although, the water samples from the different tanks of the cultivations systems were characterized by a similar content of *Pseudomonas* spp. (*p* > 0.05), the mean load of microbes in the hydroponic system was 1.03 ± 0.12 Log_10_ CFU/mL higher. In particular, hydroponic water had 1.10 ± 0.09 and 0.97 ± 0.16 Log_10_ CFU/mL more coliforms and *Enterobacteriaceae*, respectively (*p* < 0.05). Importantly, water from the fish tank had low values of coliforms, *Enterobacteriaceae* and *Pseudomonas* spp., which in high numbers perturb water quality and could result in pathogenicity for fishes. These three groups, along with the scarce quantitation of enterococci, were found in the biofilter in higher concentrations with respect to water samples from the cultivation tanks, although not significantly in that of the hydroponic system (*p* < 0.05). Similarly, in a study about the microbial populations of different sites of an aquaponic system, the biofilter was found to have the highest diversity [3,31].

### 3.6. Correlations among VOCs and Microbial Groups

**Figure 8:**
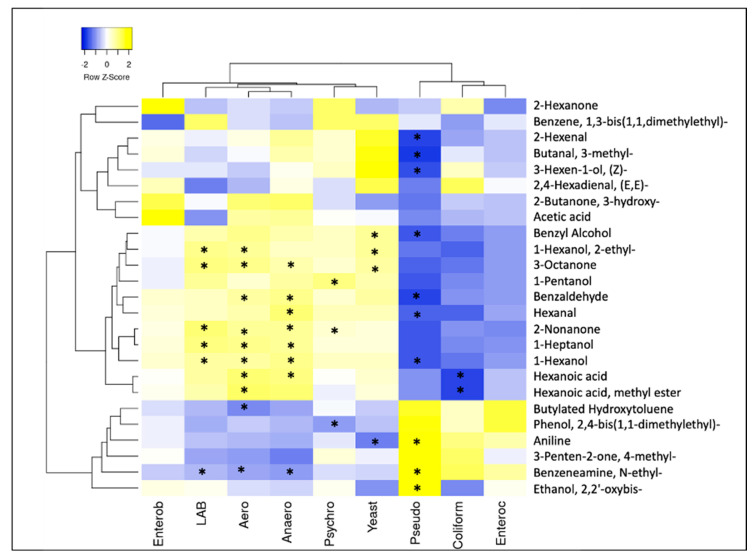
Spearman rank correlations among the VOCs and microbial groups. Enterob = *Enterobacteriaceae*; LAB = lactic acid bacteria; Aero = aerobic mesophilic bacteria; Anaero = anaerobic mesophilic bacteria; Psychro = psychrotrophic bacteria; Yeast = yeasts and molds; Pseudo = *Pseudomonas* spp.; Coliform = coliforms; Enterococ = enterococci. Left side dendrogram is identifying, by Euclidean analysis, the three major different clusters among the VOC variables. * Significance of correlations at *p* < 0.05 by Pearson test.

Spearman rank correlations (*p* < 0.05), two-way joining heatmaps and Pearson cluster analysis were performed from the comparison of two different and independently normalized datasets, derived from values of the relative quantification of VOCs and quantification of microbial targets (Figure 8). From the Pearson dendrograms, three clusters were identified; the first cluster is mainly related to leaves, the second looks more related to roots, while the third is related to the system. In Cluster 1, four VOCs out of eight were related to leaves, in particular three out of the four described CI leaves, namely, 3-hexen-1-ol, (Z)-, butanal, 3-methyl- and 2-hexenal. In fact, butanal, 3-methyl- and 2-hexenal were negatively correlated to the *Pseudomonas* spp. group, which was not found in CI leaves (*p* < 0.05). In contrast, these three variables were positively correlated, although not significantly, with the yeast group whose quantity was higher in CI leaves than in LS leaves. Birch et al. [34] found that 3-methylbutanal is among the most aromatic compounds in wheat breadcrumbs, formed by yeast metabolism. This suggested that even in our samples the presence of 3-methylbutanal was linked to the presence of yeasts on CI leaves. In Cluster 2, ten VOCs out of 11 were related to roots, in particular the eight described in CI roots, namely benzyl alcohol, 1-hexanol, 2-ethyl-, benzaldehyde, hexanal, 1-heptanol, 1-hexanol, hexanoic acid, and hexanoic acid, methyl ester. The three latter were positively correlated to both aerobic and anaerobic mesophilic bacteria (*p* < 0.05), which were greater in CI roots. Lastly, Cluster 3 was related to the system, where aniline, benzeneamine, N-ethyl- and ethanol, 2,2′-oxybis described the biofilter. Actually, each one was positively correlated (*p* < 0.05) with the *Pseudomonas* spp. group that, among the components of the system, was larger in the biofilter, while benzeneamine, N-ethyl- was negatively but not significantly (*p* > 0.05) correlated with LAB, which were not quantified in this niche. In accordance with our results, Day et al. [35] observed an accumulation of *Pseudomonas* in the biofilters of an aquaponic system during a cultivation cycle. The *Pseudomonas* spp. group accumulated in the biofilter could be derived from the roots of the plants, along which this microorganism coexisted in symbiotic association. It is assumed that this relationship can promote plant growth through the enrichment of proteins linked to energy metabolism and cell division [36].

## 4. Conclusions

This study is one of the few investigations about the principal not nitrifying microbial groups and their VOCs present at different sites of soilless cultivation systems, applying multivariate statistical analysis. In particular, this is the first time comparing the microbiological aspect of food quality and safety between either aquaponic to hydroponic systems and two different vegetable products, *L. sativa* and *C. intybus.* Besides, this work strengthened its results by indicating significance via Spearman correlations between the microbes and VOCs. The results obtained told that (i) *C. intybus* had higher microbial loads, both in the roots and the leaves; (ii) the aquaponic grown plants system had higher general loads of microbes, but surprisingly lower amounts of coliforms, with the exception of LS leaves, in comparison to hydroponic; (iii) water samples from the hydroponic tanks had higher loads of almost any microbial target found, but this water does not come in direct contact with plants; iv) the potent phytostimulant 2-nonanone was a descriptor of LS roots only and was positively correlated with higher loads of mesophilic and psychrotrophic bacteria; and v) toxic compounds, such as aniline and benzanamine, were correlated with *Pseudomonas* spp., and were the most concentrated in the biofilter, efficiently limiting their diffusion in other compartments of the system. There is a special caution pertaining to the microbial safety of food products coming from aquaponic cultivation systems due to their close contact to fish wastes dispersed in the water; however, according to this preliminary work, the aquaponics phyllosphere apparently had a microbial quality similar to hydroponic ones. Nowadays, precision agriculture is the approach towards a modern agriculture. It is based on specific strategies combining agronomic approaches and supply chain solutions to obtain sustainable, safe and healthy food. The contribution of this work may represent the first step in providing a simple method, based on the assessment of the VOCs in a whole ecosystem, as a tool to predict the microbial quality of hydroponic- and aquaponic-cultivated vegetables, eventually deciding the different food product destination (fresh or cooked) to preserve their safety and nutritional value. Nevertheless, the specific role of microbes must be more deeply addressed through the assessment of population genomics, in order to point out more cause–effects issues, to monitor and control any environment of this representative holobiome system.

## Data Availability

Not applicable.

## Supplementary Materials

The following are available online at https://www.mdpi.com/2076-2607/9/3/580/s1, Table S1: Sample descriptions. Table S2: Microbiology methods. Table S3: Microbial quantification of the cultivation apparatus at the baseline. Figure S1: Quantification heatmap of relative abundances of volatile organic compounds (VOCs) of samples. Figure S2: MANOVA (*p* < 0.01) for contributions of categorized independent variables on VOCs descriptors.
