# Supplementary material for: Plant Volatiles of Lettuce and Chicory Cultivated in Aquaponics Are Associated to Their Microbial Community
Source: Microorganisms. 2021 Mar 12;9(3):580. doi: 10.3390/microorganisms9030580 (PMC7998580; doi:10.3390/microorganisms9030580)
Supplement: Supplementary file 1 [file microorganisms-09-00580-s001.pdf]

Table S1. Sample descriptions

Sample	Description
LS Leaves A	*Lactuca sativa* (L.) var. Salanova grown in aquaponic system*
LS Leaves H	*Lactuca sativa* (L.) var. Salanova grown in hydroponic system*
CI Leaves A	*Cichorium intybus* (L.) grown in aquaponic system*
CI Leaves H	*Cichorium intybus* (L.) grown in hydroponic system*
LS Roots A	*Lactuca sativa* (L.) var. Salanova roots grown in aquaponic system
LS Roots H	*Lactuca sativa* (L.) var. Salanova roots grown in hydroponic system
CI Roots A	*Cichorium intybus* (L.) roots grown in aquaponic system
CI Roots H	*Cichorium intybus* (L.) roots grown in hydroponic system
Fish	Fillet of Nile tilapia (*Oreochromis niloticus* L.)**
Biofilter	Biofilter water
Fish tank	Fish tank water
Water A	Cultivation water of aquaponic system
Water H	Cultivation water of hydroponic system

*plants were sampled after 30 days of development; **fishes were sampled after 4 months of growth.

Table S2. Microbiology methods

Culture media	ISO Methodology*	Microbial group	Time	Conditions	Temperature
Violet Red Bile Glucose Agar (VRBGA)	[1]	*Enterobacteriaceae*	48 h**	Anaerobiosis#	37 °C
Violet Red Bile Agar (VRBA)	[2]	Coliforms	24 h	Aerobiosis	30 °C
Plate Count Agar (PCA)	[3]	Mesophilic aerobic bacteria	48 h	Aerobiosis	30 °C
Plate Count Agar (PCA)	[4]	Mesophilic anaerobic bacteria	48 h	Anaerobiosis	30 °C
Plate Count Agar (PCA)	[5]	Psychrotrophic bacteria	10 d*#	Aerobiosis	7 °C
De Man, Rogosa, Sharp (MRS)	[6]	Lactic acid bacteria	48 h	Anaerobiosis	37 °C
Slanetz and Bartley	[7]	Enterococci	48 h	Aerobiosis	30 °C
Sabouraud Dextrose Agar (SAB) and CAF##	[8]	Fungi and yeasts	48 h	Aerobiosis	30 °C
Pseudomonas Agar Base (PAB) with Pseudomonas CFC Supplement (SR0103)	[9]	*Pseudomonas* spp.	48 h	Aerobiosis	30 °C

*Adapted from ISO; **h = hours; #Anaerobiosis was achieved with anaerobic jars and catalyst (Thermo Fisher Scientific, Waltham, MA, USA); *# d = days; ##CAF = Chloramphenicol (0.05 mg/L).

Table S3. Microbial quantification of the cultivation apparatus at the baseline.

**Microbial target**	**Quantifications (Log_{10} CFU/mL)**			
	**Biofilter**	**Fish tank**	**Water A***	**Water H****
Enterobacteriaceae	5.33 ± 0.11	4.04 ± 0.11	3.08 ± 0.17	< 1
Coliforms	4.79 ± 0.14	3.69 ± 0.18	3.35 ± 0.10	< 1
Mesophilic aerobic bacteria	5.29 ± 0.21	3.55 ± 0.12	3.41 ± 0.12	1.88 ± 0.09
Mesophilic anaerobic bacteria	5.14 ± 0.24	3.49 ± 0.21	2.31 ± 0.09	< 1
Psychrotrophic bacteria	5.77 ± 0.32	3.62 ± 0.28	4.01 ± 0.28	1.38 ± 0.08
Lactic acid bacteria	< 1	< 1	< 1	< 1
Enterococci	< 1	< 1	< 1	< 1
Fungi and yeasts	< 1	< 1	< 1	< 1
Pseudomonas spp.	4.80 ± 0.22	3.53 ± 0.18	2.71 ± 0.14	1.54 ± 0.10

*A = aquaponic system; **H = hydroponic system

Figure S1. Quantification heatmap of relative abundances of volatile organic compounds (VOCs) of samples*.

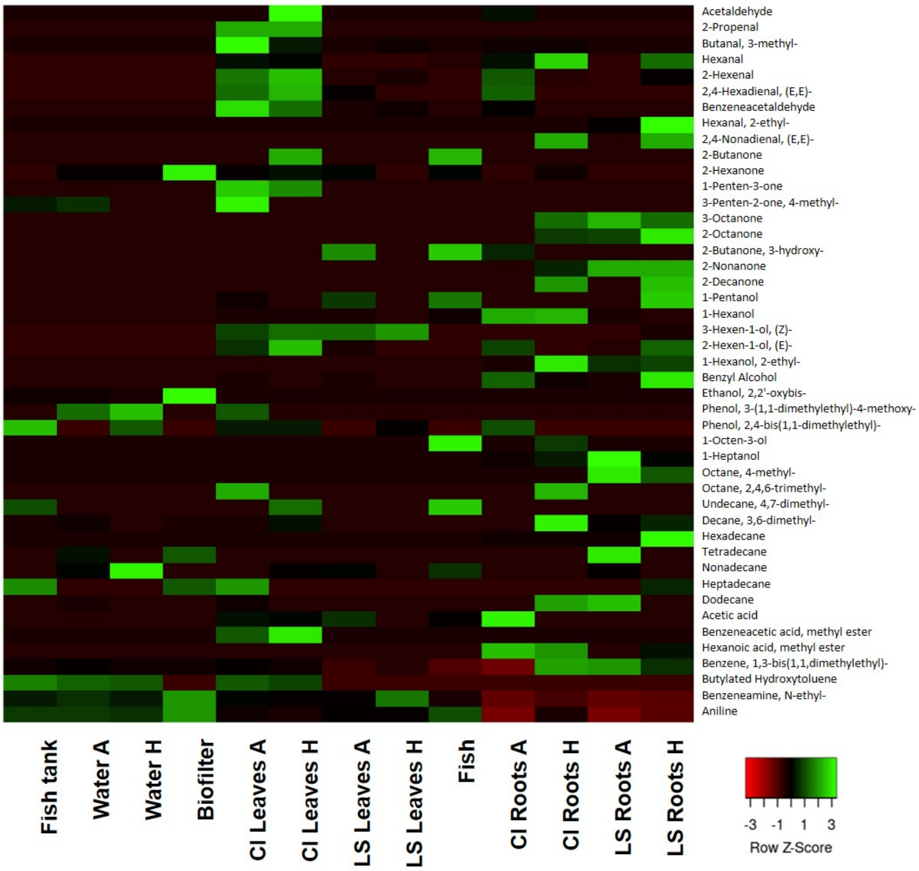

*For sample codes see table S1.

Figure S2. MANOVA ($p < 0.01$) for contributions of categorized independent variables on VOCs descriptors.

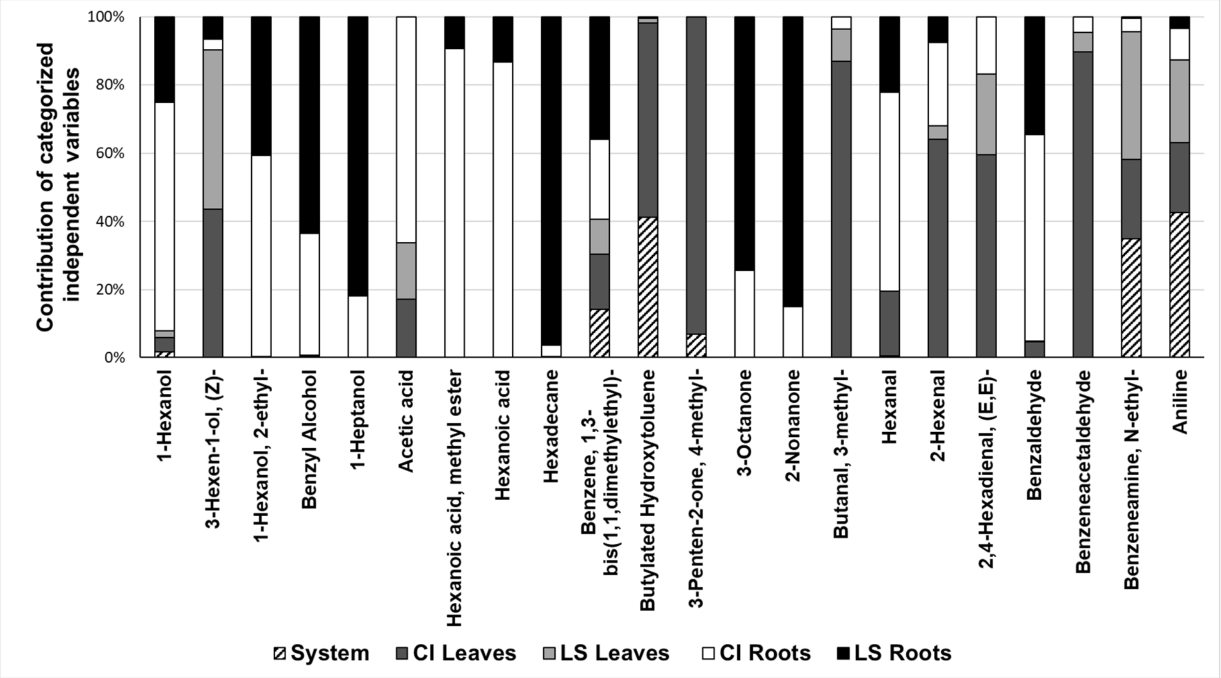

1. International Organization for Standardization. *Horizontal Method for Detection and Enumeration of Enterobacteriaceae Part 1: Colony-Count Method*; ISO 21528-1:2004; ISO: Geneva, Switzerland, 2004.
2. International Organization for Standardization. *Horizontal Method for the Detection and Enumeration of coliforms–Colony count Method*; ISO 4823:2006; ISO: Geneva, Switzerland, 2006.
3. International Organization for Standardization. *Microbiology of the Food Chain–Horizontal Method for the Enumeration of Microorganisms–Part 1: Colony Count at 30 Degrees C by the Pour Plate Technique*; ISO 4833-1:2013; ISO: Geneva, Switzerland, 2013.
4. International Organization for Standardization. *Microbiology of Food and Animal Feeding Stuffs—Horizontal Method for the Enumeration of Sulfite-Reducing Bacteria Growing under Anaerobic Conditions*; ISO 15213:2003; ISO: Geneva, Switzerland, 2003.
5. International Organization for Standardization. *Microbiology of the Food Chain—Horizontal Method for the Enumeration of Psychrotrophic Microorganisms*; ISO 17410:2019, ISO: Geneva, Switzerland, 2019.
6. International Organization for Standardization. *Milk Products—Enumeration of Presumptive Lactobacillus Acidophilus on a Selective Medium—Colony-Count Technique at 37 °C*; ISO 20128:2006; ISO: Geneva, Switzerland, 2006.
7. *Nordic Committee on Food Analysis*; Leaflet: Westwood, MA, USA, 1968.
8. International Organization for Standardization. *Microbiology of Food and Animal Feeding Stuffs—Horizontal Method for the Enumeration of Yeasts and Moulds*; ISO 21527:2008; ISO: Geneva, Switzerland, 2008.
9. International Organization for Standardization. *Meat and Meat Products—Enumeration of Presumptive Pseudomonas spp*; ISO 13720:2010; ISO: Geneva, Switzerland, 2010.
